# Can heat waves change the trophic role of the world’s most invasive crayfish? Diet shifts in *Procambarus clarkii*

**DOI:** 10.1371/journal.pone.0183108

**Published:** 2017-09-05

**Authors:** Bruno M. Carreira, Pedro Segurado, Anssi Laurila, Rui Rebelo

**Affiliations:** 1 cE3c Centre for Ecology Evolution and Environmental Changes, Faculdade de Ciências da Universidade de Lisboa, Lisboa, Portugal; 2 Animal Ecology/ Department of Ecology and Genetics, Uppsala University, Uppsala, Sweden; 3 Centro de Estudos Florestais, Instituto Superior de Agronomia da Universidade de Lisboa, Lisboa, Portugal; University of Connecticut, UNITED STATES

## Abstract

In the Mediterranean basin, the globally increasing temperatures are expected to be accompanied by longer heat waves. Commonly assumed to benefit cold-limited invasive alien species, these climatic changes may also change their feeding preferences, especially in the case of omnivorous ectotherms. We investigated heat wave effects on diet choice, growth and energy reserves in the invasive red swamp crayfish, *Procambarus clarkii*. In laboratory experiments, we fed juvenile and adult crayfish on animal, plant or mixed diets and exposed them to a short or a long heat wave. We then measured crayfish survival, growth, body reserves and Fulton’s condition index. Diet choices of the crayfish maintained on the mixed diet were estimated using stable isotopes (^13^C and ^15^N). The results suggest a decreased efficiency of carnivorous diets at higher temperatures, as juveniles fed on the animal diet were unable to maintain high growth rates in the long heat wave; and a decreased efficiency of herbivorous diets at lower temperatures, as juveniles in the cold accumulated less body reserves when fed on the plant diet. Heat wave treatments increased the assimilation of plant material, especially in juveniles, allowing them to sustain high growth rates in the long heat wave. Contrary to our expectations, crayfish performance decreased in the long heat wave, suggesting that Mediterranean summer heat waves may have negative effects on *P*. *clarkii* and that they are unlikely to boost its populations in this region. Although uncertain, it is possible that the greater assimilation of the plant diet resulted from changes in crayfish feeding preferences, raising the hypotheses that i) heat waves may change the predominant impacts of this keystone species and ii) that by altering species’ trophic niches, climate change may alter the main impacts of invasive alien species.

## Introduction

The metabolic processes and the stoichiometric balance of ectotherms are both strongly influenced by temperature. This raises concern for the stability of freshwater ecosystems, because climate change is expected to have particularly pervasive effects in these ecosystems [1–2], where approximately 99% of the species are ectotherms [3]. Moreover, recent research shows temperature may play a determining role on nutrient acquisition by omnivorous ectotherms [4]. As such, considering the current climatic projections, a better understanding of temperature and nutrient interactions is crucial to develop realistic predictions of the ecological responses to climate change [5]. Invasive alien species (IAS) are an important driver of global change in freshwater ecosystems [6–7], and are expected to have its greatest impact in Mediterranean biomes, which have long been isolated [8–9]. Importantly, climate change may interact with IAS and cause severe damage to freshwater communities. The removal or weakening of environmental barriers by climate change may provide IAS with new suitable habitats and facilitate their expansion due to the high connectivity of freshwater ecosystems [10–12].

Overall, climate change may not only increase population size and geographic range of ectothermic IAS, but also change the nature of their trophic impacts as they optimize nutrient intake for higher temperatures. Temperature influences nutrient intake in omnivorous ectotherms through imbalanced effects on various metabolic aspects that scale differently with temperature. In general, these effects favor the assimilation of plant diets (carbohydrate-rich) at higher temperatures. By promoting a greater increase in feeding and gut passage rates than in assimilation [13–14], higher temperatures favor the assimilation of smaller and structurally less complex nutrients such as carbohydrates over complex nutrients such as proteins, which take longer to digest. In fact, the protein to carbohydrate assimilation ratio of crayfish was found to shift to a greater assimilation of carbohydrates at higher temperatures [15]. Alternatively, increasing the consumption of carbohydrate-rich plant diets may help ectotherms to cope with the greater energetic demands at higher temperatures. Due to the stronger effect on catabolic than on anabolic processes, higher temperatures promote a greater increase in respiration than in growth and increase the demand for carbon over nitrogen [16–17]. Additionally, increasing the consumption of fast energy sources mitigates the effects of decreased digestion efficiency at higher temperatures resulting from the greater increase in metabolic than in feeding rates [18–19]. This evidence suggests that omnivorous ectotherms should optimize energy intake by avoiding protein-rich diets and increase herbivory at higher temperatures, which has recently been proven to hold true in different ectotherm taxa [20–23]. This relationship between the feeding preferences of ectothermic organisms and temperature, potentially leading to a greater herbivory by omnivorous ectotherms, should extend to IAS. Therefore, through its effects on the individual metabolism, climate change may alter the trophic position of ectothermic IAS and their impacts on ecosystems.

Many of the most abundant IAS in Europe are native to tropical or subtropical regions, where climatic conditions are substantially different [24]. The more similar climatic conditions in the new environments are to those in the native distribution range of the invasive species, the more likely invasions are to succeed [25–26]. As cold tolerance is one of the most important traits shaping biological range distributions [27], the higher temperatures arising from climate change may weaken the constraints imposed by cold temperatures on cold-limited IAS [28–30]. Ranking among world’s worst IAS in terms of ecological and economic impacts, the omnivorous red swamp crayfish *Procambarus clarkii* (Girard, 1852) is native to warm environments in the central south of the USA and the northeast of Mexico, where its optimum growth temperature ranges from 20 to 27°C [31]. Although well-established in the Iberian Peninsula, the Iberian populations experience average minimum temperatures 2 to 5°C lower than in the native range, with spring and summer temperatures being 7 to 8°C lower. Projections indicate a rise in the winter minima up to 3°C in the Iberian Peninsula [32–34], bringing climatic conditions closer to those in the native range of *P*. *clarkii*. Furthermore, projections also indicate that extreme climatic events such as heat waves are likely to become longer, more frequent and intense [35]. These strong perturbations may further benefit *P*. *clarkii* and other cold-limited IAS, since extreme climatic events often create resource pulses and reduce the communities’ biotic resistance to invaders [36–38].

In this study, we investigated the effects of simulated heat waves on *P*. *clarkii*. Given it is both an omnivorous ectotherm and a cold-limited IAS, predicted climate changes may affect the impact of *P*. *clarkii* on aquatic communities in multiple ways. We fed juvenile and adult crayfish with animal-based, plant-based or mixed diets and exposed them to temperature treatments simulating either the current common short heat waves or long heat waves expected to become more frequent in the future. We recorded crayfish survival and life-history traits (growth rate, body reserves and Fulton’s index) and reconstructed the dietary choices of crayfish fed on mixed diets using stable isotopes. We predicted that in the heat wave treatments this subtropical crayfish should: (1) decrease performance on the animal diet; (2) improve performance on the plant diet; (3) increase herbivory on the mixed diet; (4) increase performance relative to colder treatments.

## Materials and methods

### Collection and maintenance

Crayfish collection was carried out under the permit no. 211/2014/CAPT from Instituto da Conservação da Natureza e das Florestas. Crayfish were captured with dip-net sweeps in rice field ditches near Samora Correia (38°52’N, 8°51’W), before the main reproductive episode in the fall [39]. Adults were collected on the 30^th^ of May 2013 (total body length: 60-90mm) and immature juveniles from the previous autumn cohort on the 25^th^ of July 2013 (total body length: 40 to 45mm), when average water temperatures range from 18 to 23°C and maximum water temperature averages 25°C [39]. Crayfish were acclimatized for two months at room temperature (ca. 20°C) under 12L:12D photoperiod and fed commercial fish food every other day. Adults were maintained individually in 1.5L aquaria and entered the experiment on the 31^st^ of July 2013, while juveniles were maintained in groups of 15 individuals in 5L aquaria and entered the experiment on 1^st^ of October 2013.

### Experimental procedures

At the start of the experiments individuals were blotted dry in paper towel, weighed and measured (carapace length–CL; post orbital carapace length–POCL). Crayfish were transferred to individual aerated aquaria (1.5L) placed in water baths, following a fully factorial experimental design with diet (three levels) and temperature (four levels) as factors (see section *Diet and Temperature*). We balanced the replicates’ sex ratio and randomly assigned each treatment combination to ten juveniles (5 Females:5 Males) and seven adult crayfish (4 Females:3 Males). Crayfish position in the water baths was randomized within treatments after each provision of food. Individuals were allowed to feed overnight every other day, and food remains were removed along with water renewal in the following morning. Experiments lasted for two months to exceed the half-life of ^13^C and ^15^N turnover rates (ca. 6–8 weeks for 10-100g organisms at 10–30°C; [40]).

Deaths throughout the experiments were recorded and crayfish were weighed and measured at the end of the experiment. Growth rate was calculated as weight variation (mg) divided by the experiment duration (days); Fulton’s index, the species expected weight at a given length, was determined with CL [41]. Individuals were then euthanized by rapid freezing at -18°C, to avoid contamination of the isotopic signature with euthanizing agents. We estimated body reserves allocated to maintenance (hepatopancreas) and to reproduction (gonads) together, expressing it as a percentage of the body mass in both sexes, because hepatopancreas and gonads were difficult to separate in defrosted male crayfish. Abdominal muscle samples were prepared for stable isotope analysis: dried at 60°C for 24 h, ground to fine powder with mortar, cleansed of storage lipids with chloroform-methanol (2:1; [42]) and re-dried at 60°C for 24h.

### Diet and temperature

We tested three experimental diets, all offered *ad libitum*: Animal diet (A)–composed of defrosted Chironomidae larvae, favored by *P*. *clarkii* and common in temporary ponds and rice fields [43–44]; Plant diet (P)–composed of defrosted stalks of *Juncus heterophyllus*, an emergent macrophyte abundant in Mediterranean temporary ponds and favored by *P*. *clarkii* [45]; and Mixed diet (M)–composed of both food items in diets A and P offered in similar proportions of fresh mass.

**Fig 1:**
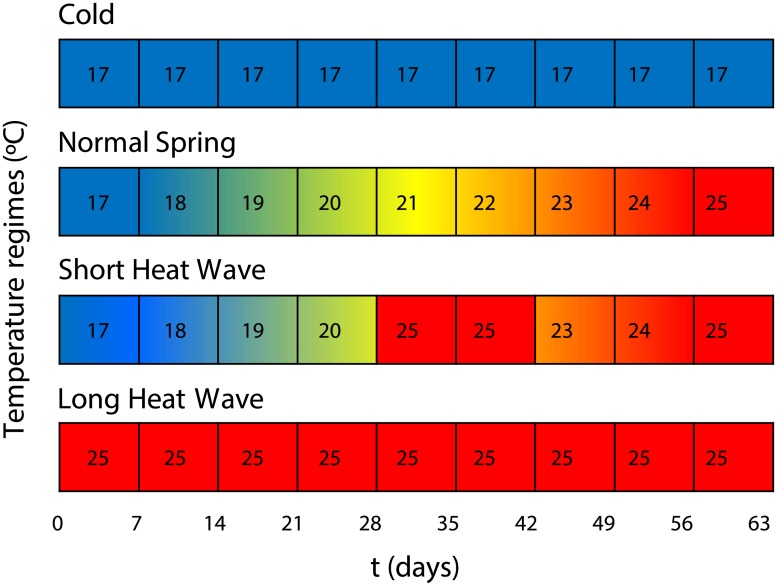
Schematic representation of the temperature treatments. Temperature variation over time in each experimental treatment.

The experiments included four temperature treatments (Fig 1): Cold (C)–constant temperature of 17°C; Normal Spring (NS)–temperature was gradually increased from 17 to 25°C, at the rate of 1°C per week (average 21°C); Short Heat Wave (SHW)–similar to NS, but on day 28 crayfish were exposed to a two-week heat wave of 25°C, after which temperature was decreased to 23°C (going back to the same regime as in NS); and Long Heat Wave (LHW)–constant temperature of 25°C. The two-week heat wave (SHW) aimed to simulate the maximum duration of current spring heat waves in the southwest of Portugal, which typically last for one to two weeks (B. M. Carreira, *unpubl*. *data*). The duration of these heat waves was estimated with a 10-year data set of air temperatures (2002–2012) and following the heat have definition by Frich et al. [46] stating that a heat wave occurs when the daily maximum temperature exceeds the average maximum temperature by 5°C for more than five consecutive days (reference period: 1961–1990). The two-month heat wave (LHW) aimed to simulate extremely long heat waves, such as the one that afflicted Europe in 2003, an extreme climatic event that is expected to become more frequent in the future [32–33].

### Isotope analysis

Stable isotope ratios (^13^C/^12^C, ^15^N/^14^N) and elemental analyses (C:N) were determined by continuous flow isotope mass spectrometry (CF-IRMS) [47], on a Hydra 20–22 (Sercon, UK) stable isotope ratio mass spectrometer, coupled to a EuroEA (EuroVector, Italy) elemental analyzer for online sample preparation by Dumas-combustion. The standards for carbon isotope ratio were IAEA-CH6 and IAEA-CH7, and the standards for nitrogen isotope ratio were IAEA-N1 and USGS-35. δ^13^C results were referred to PeeDee Belemnite (PDB) and δ^15^N to Air. Estimated precision of the isotope ratio analysis was ≤ 0.2‰ (6 to 9 replicates of laboratory standard material in every batch analysis).

### Statistical analyses

We used stable isotope mixing models to obtain time-integrated estimates of the diet choices of the crayfish maintained on the mixed diet. The Stable Isotope Analysis package in R (SIAR [48]) fits a Bayesian model to the proportions of the sources contributing to the consumers’ isotopic signature and generates probability distribution functions for the proportions of animal and plant material (10.000 iterations). These models incorporate variability in the isotopic signature and elemental composition of the sources, and in the trophic enrichment factors [49]. Conforming to the standard procedures, we specified the isotopic signatures and elemental ratio of the sources, which differed substantially in the δ^13^C and C:N ratios (Table 1), but we built separate models for each life stage. Furthermore, we specified specific trophic enrichment factors for each combination of diet and temperature conditions, which were estimated using the isotopic signatures of crayfish fed on single diets (animal or plant diet) in the different temperature regimes.

**Table 1:** Isotopic signature and elemental ratio of the experimental diets.

	Animal diet	Plant diet
**δ** ^**13**^**C**	-24.61 ± 0.13	-28.63 ± 0.19
**δ** ^**15**^**N**	5.09 ± 0.92	4.41 ± 0.44
**C:N**	3.79 ± 0.05	23.69 ± 11.52

Carbon (δ ^13^C) and nitrogen (δ ^15^N) isotopic signatures, and C:N elemental ratio of the food items composing the animal and the plant diets provided to juvenile and adult *Procambarus clarkii* (average ± standard deviation).

Treatment effects on the survival of adult crayfish were tested with the Cox proportional hazards regression model in the survival package of R software. The model included data from additional individuals removed immediately after the short heat wave and used in a different study. We used general linear models (GLMs), performed on STATISTICA 12.6.255.0 (StatSoft), to test for temperature and diet effects (fixed factors) and their interactions on growth rate, body reserves and on Fulton’s index, including initial POCL as a covariate. Post hoc pairwise comparisons were corrected for multiple comparisons (Bonferroni’s test).

## Results

### Juveniles

**Fig 2:**
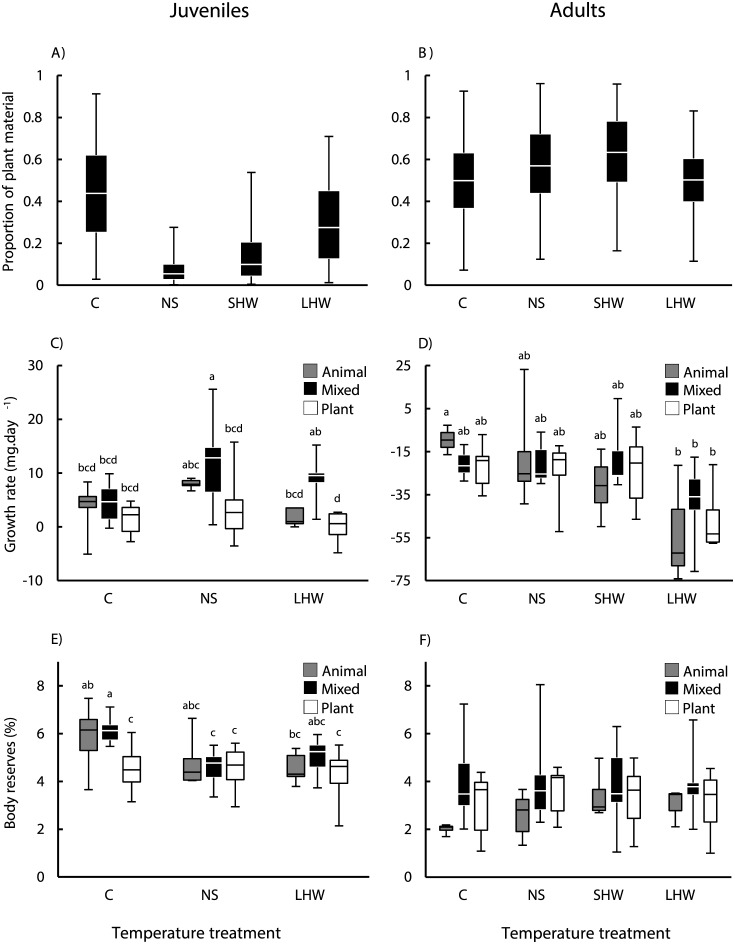
Temperature treatment effects on the assimilation of plant material, growth and body reserves of juvenile and adult *Procambarus clarkii*. Proportion of plant material assimilated, growth rate (mg.day^-1^) and body reserves (%) of juvenile (a, c, e) and adult (b, d, f) *P*. *clarkii*, respectively, in the different temperature treatments: Cold (C), Normal Spring (NS), Short Heat Wave (SHW), and Long Heat Wave (LHW). Note: The boxes show the median and the 25^th^– 75^th^ percentiles and the whiskers indicate the 2.5^th^– 97.5^th^ percentiles (a, b) or the minimum and the maximum values (c, d, e, f).

Juveniles on the mixed diet were mostly carnivorous and assimilated an average median proportion of plant material of 0.217 (Fig 2a). Despite of the overlap in the 95% Bayesian Credible Intervals (BCI) for the proportion of plant material assimilated in the different temperature regimes, the median and the 25^th^-75^th^ percentiles of the probability distributions show clear tendencies. Juveniles were most herbivorous in the cold treatment, for which the median proportion of plant material assimilated amounted to 0.438 (BCI = 0.029–0.913), and carnivorous in the normal spring treatment, for which the median proportion of plant material assimilated amounted only to 0.055 (= 0.003–0.276; Fig 2a). In the short heat wave the median proportion of plant material assimilated by juveniles increased to 0.099 (BCI = 0.004–0.538) and in the long heat wave it increased even more to 0.275 (BCI = 0.012–0.709; Fig 2a).

Although none of the juvenile crayfish died directly as a result of the experimental treatments, a spoiled batch of chironomid larvae caused high mortality in some treatments, roughly a week before the start of the short heat wave. This incident killed five juveniles (50%) in both the “Normal Spring × Animal diet” and the “Long Heat Wave × Animal diet” treatments; and all juveniles in the “Short Heat Wave × Animal diet” treatment. This forced us to discard the Short Heat Wave from the general linear models used to detect effects on growth rate and body reserves, and to run the models with fewer observations in the “Normal Spring × Animal diet” and the “Long Heat Wave × Animal diet” treatments (N = 5).

Growth on the mixed diet was two times greater than on the animal diet and five times greater than on the plant diet (Table 2; Fig 2c). Growth in normal spring was two times greater than in cold and long heat wave treatments (Table 2; Fig 2c). However, a significant diet × temperature interaction showed that while in the cold there was no diet effect on growth, in the normal spring and in the long heat wave growth on the mixed diet was higher than on the plant diet (Table 2; Fig 2c).

**Table 2:** General linear models—Juveniles.

	Growth rate			Body reserves			Fulton’s index		
Factors	*df*	F	*P*	*df*	F	*P*	*df*	F	*P*
Diet	2, 70	21.88	<0.001^a^	2, 70	7.76	<0.001^a^	2, 71	5.28	<0.01^a^
Temperature	2, 70	10.43	<0.001^a^	2, 70	9.83	<0.001^a^	2, 71	5.78	<0.01^a^
Diet × Temperature	4, 70	3.17	<0.05^a^	4, 70	2.63	<0.05^a^	4, 71	0.41	0.801
POCL (covariate)	1, 70	4.63	<0.05^a^	1, 70	0.36	0.551			

General linear models and statistics for growth rate, body reserves and Fulton’s index of juvenile *Procambarus clarkii*.

^a^*P* values <0.05.

Body reserves on the animal and mixed diets were 16% greater than on the plant diet (Table 2; Fig 2e). In general, body reserves in the cold treatment were 19% greater than in the normal spring and long heat wave treatments (Table 2; Fig 2e). A significant diet × temperature interaction showed that unlike in the other temperature treatments, in the cold treatment body reserves on the plant diet were lower than on the other diets (Table 2; Fig 2e).

Overall, Fulton’s index for juveniles at the start of the experiment averaged 0.277 ± 0.020, with no statistical differences among treatments. At the end of the experiment Fulton’s index on the animal diet (0.217 ± 0.02) was greater than on the plant diet (0.199 ± 0.017; Table 2). Fulton’s index in the cold and in the normal spring (0.212 ± 0.020) was higher than in the long heat wave (0.195 ± 0.017; Table 2).

### Adults

On the mixed diet adults fed similarly of both food items and the average median proportion of plant material assimilated amounted to 0.550 (Fig 2b). As the large overlap in the 95% Bayesian Credible Intervals (BCI) for the proportion of plant material assimilated in the different temperature regimes also extended to the 25^th^-75^th^ percentiles of the distributions, we comment only on tendencies in the median. The median proportion of plant material assimilated by adults was lowest in the cold (0.499; BCI = 0.072–0.925) and in the long heat wave treatments (0.502; BCI = 0.115–0.831). In the normal spring, the median proportion of plant material assimilated increased to 0.569 (BCI = 0.123–0.961) and in the short heat wave it increased even more to 0.633 (BCI = 0.164–0.959; Fig 2b).

Survival on both the plant and the mixed diets was high, 93% and 76% respectively, but on the animal diet it dropped significantly to only 50% (χ^2^ = 20.09; p <0.001). Survival in the cold treatment (95%) was higher than in the other temperature treatments for which survival ranged from 62% in normal spring to 81% in long heat wave (χ^2^ = 11.64; p <0.01). We found no significant diet × temperature interaction affecting the survival of adult crayfish (χ^2^ = 8.54; p = 0.201).

Adults experienced a general loss of body mass during the experiment, which was similar across the different diet treatments (Table 3; Fig 2d). The loss of body mass was more pronounced in the long heat wave treatment, where crayfish lost ca. two times more mass than in the other temperature treatments (Table 3; Fig 2d).

**Table 3:** General linear models—Adults.

	Growth rate			Body reserves			Fulton’s index		
Factors	*df*	*df*	F	*df*	F	*df*	F	F	*P*
Diet	2, 63	0.02	0.979	2, 63	4.63	<0.05^a^	2, 64	0.34	0.871
Temperature	3, 63	9.374	<0.001^a^	3, 63	0.34	0.797	3, 64	0.42	0.715
Diet × Temperature	6, 63	0.99	0.442	6, 63	0.35	0.908	6, 64	0.38	0.892
POCL	1, 63	13.08	<0.001^a^	1, 63	2.34	0.131			

General linear models and statistics for growth rate, body reserves and Fulton’s index of adult *Procambarus clarkii*.

^a^*P* values <0.05.

Body reserves on the mixed diet were 44% greater than on the animal diet (Table 3; Fig 2f). Temperature had no significant effect on adult on body reserves and there was no significant diet × temperature interaction.

Overall, Fulton’s index for adults at the start of the experiment averaged 0.294 ± 0.026, with no statistical differences among treatments. Fulton’s index at the end of the experiment, averaged 0.241 ± 0.028 and was not significantly affected by diet or temperature (Table 3).

## Discussion

To our knowledge, this is one of the first studies to investigate the role of temperature on nutrient acquisition by omnivorous ectotherms, and the first to indicate potential temperature-driven changes in the consumptive impacts of different life stages of a well-known widely distributed invasive alien species. We found that heat waves may affect *Procambarus clarkii* in multiple ways due to its nature as both an ectotherm and an invasive alien species. The ongoing climatic changes may potentially increase the herbivory of this species, particularly in juveniles, which showed a trend to increase the assimilation of plant material in the heat waves, similar to other ectotherm species experiencing higher temperatures. By increasing herbivory *P*. *clarkii* may optimize nutrient intake and better cope with the greater energetic demands at higher temperatures. Additionally, the longer heat waves arising with climate change may have a negative impact on *P*. *clarkii*, as the performance of both juvenile and adult crayfish decreased in the long heat wave. Thus, besides the detrimental effects on the Mediterranean populations of *P*. *clarkii*, the trends in our results suggest that heat waves may partially shift the most predominant consumptive impact of the juvenile cohorts, decreasing predation upon macroinvertebrates and increasing herbivory upon macrophytes and crops.

### *Procambarus clarkii*—Omnivorous ectotherm

The contrasting temperature effects found in crayfish on the single diets provide some support to the change in the relative quality of diet with temperature described in recent works [4,22,50]. The growth results of both juveniles and adults show a decreased performance of the animal diet in the long heat wave, suggesting a lower nutritional value of this diet at higher temperatures. In fact, the most detrimental effects of the animal diet and elevated temperatures were the lower adult survival, which may indicate that adults were unable to meet the higher energetic demands imposed by their greater size when compared to juveniles, which suffered no apparent effect on survival. The performance on plant diet changed very little across temperature treatments and we found no evidence for it to increase in the heat wave treatments. However, in the cold treatment juvenile body reserves on the plant diet were lower than on the other diets, suggesting a decreased performance of the plant diet at lower temperatures, also found in vertebrate ectotherms [4]. The relatively minor changes in crayfish performance on the animal and plant diets across temperature regimes may stem from limitations in our experimental setup that aimed to replicate conditions in nature during late spring and early summer. This led to narrow temperature range that included the temperatures for which the documented shift in the protein to carbohydrate assimilation ratio is smallest [15].

Overall, the greater average median proportion of plant material assimilated by adult *P*. *clarkii* compared to juveniles is consistent with the biology of the species, since the ontogenetic shift in its feeding preferences is well documented in the literature, juveniles being mostly carnivorous and adults mostly herbivorous or detritivorous [51]. However, the lower survival on the animal diet suggests that high herbivory in adult crayfish may provide a fast source of energy, key in coping with the higher energetic demands at greater body sizes. Against our prediction, juveniles assimilated the highest proportion of plant material in the cold treatment rather than in the long heat wave. This striking response, absent in adult crayfish, suggests juveniles may have experienced some degree of thermal stress in the cold treatment (see below). Nevertheless, the tendency in the assimilation of plant material in the other temperature regimes agreed with our prediction and the temperature-induced changes in the relative quality of diets, while small, may explain the assimilation shifts observed. Like other ectotherms, juvenile *P*. *clarkii* increased the assimilation of plant material in the heat waves, when comparing with the normal spring [4]. Even though the low Fulton´s index and body reserves on the plant diet suggest this would be a maladaptive shift, the greater assimilation of plant material allowed juveniles on the mixed diet to maintain high growth rates in the long heat wave. To a lesser extent, the influence of temperature on nutrient acquisition was also noticeable in adults. In comparison to the cold treatment, the assimilation of plant material increased in the normal spring and even more in the short heat wave, but with no apparent costs or benefits in any of the cases. Consequently, the changes projected for the Mediterranean regarding the frequency, duration and intensity of heat waves may prompt a shift in the burden of its predatory and herbivory impacts, as macroinvertebrates currently constitute a considerable proportion of *P*. *clarkii*’s diet, especially at the juvenile stage [39,52].

The role of temperature in modulating the nutrient acquisition and the feeding preferences of ectotherms from different taxonomic groups was uncovered only recently [4,18,20–22], even though other studies found no evidence of such influence [53–54]. Our study shows a tendency in a species from yet another taxonomic group for higher temperatures to increase the assimilation of plant diets, underscoring that this may be a general and consistent pattern in ectotherms. However, the true extent of the temperature influence on nutrient acquisition may be more complex and extend beyond the differential demand for carbon and nitrogen, as a recent study showed food quality to change with temperature depending on its phosphorous content [50]. Furthermore, other responses to the physiological stress imposed by low or high temperatures may superimpose to diet regulation as a function of temperature and the relative quality of the diets. For example, the surprisingly high assimilation of plant material by the juveniles in the cold treatment, also reported in the larvae of the Iberian painted frog [4], may be one of these cases. We believe this response may have been triggered by thermal stress induced by the cold treatment and the need to maintain the fluidity of cell membranes at low temperatures [55–56], since plant diets are richer in polyunsaturated lipids, and their particular molecular structure increases membrane fluidity. Hence, despite the low performance of plant diets at low temperatures, the high proportion of plant material assimilated in the cold may have helped the mostly carnivorous juveniles to maintain performance at 17°C. Interestingly, this is supported by the results in adults, which having assimilated a high proportion of plant material in all treatments did not display a similar response. On the opposite end, the unexpected lower assimilation of plant material by adults in the long heat wave seems to be consistent with the increased carnivory reported for *P*. *clarkii* in the summer [57]. Absent in juveniles, which were mostly carnivorous, this response may be related to a greater nitrogen demand at higher temperatures and the synthesis of heat shock proteins, and these benefits may outweigh the low performance of animal diets at very high temperatures.

### *Procambarus clarkii*—Invasive alien species

Contrary to our expectations, we found no evidence that heat waves improve the performance of this subtropical invader, instead the long heat wave decreased performance of both life stages. Although optimal growth in the native distribution range occurs at 20–27°C [15], juvenile growth in the long heat wave (25°C) was lower than in the normal spring treatment (17–25°C). Fulton’s index for juveniles in the long heat wave (see Results) was lower than in wild Portuguese populations (0.23 ± 0.03; [41]), suggesting the increase in size was not accompanied by a proportional increase in weight. Similarly, performance in adults also decreased in the long heat wave, for which weight loss was greater than in the other treatments. Weight loss typically occurs when crayfish are kept out of their optimum temperatures for extended periods and could indicate physiological stress [15]. However, since Fulton’s index at the end of the experiment (0.24 ± 0.03) was within normal values for wild *P*. *clarkii* (0.23 ± 0.03; [41]), we believe this may have partially resulted from a lower quality of the experimental diets in comparison with the commercial fish food that elevated Fulton’s index at the start of the experiment above normal levels. Furthermore, the lower survival rates experienced by adults in all temperature regimes but the cold treatment indicates that the increase in catabolism (i.e. respiration) imposed by elevated temperatures may have detrimental effects on this life stage. Given that respiration scales with body mass, its detrimental effects should become more apparent in adults, whose average body mass was six times greater than that of juveniles. Despite realistically covering water temperatures at the collection sites, our experimental setup may have exaggerated heat wave effects, as logistic constraints did not allow cyclical dial variation, which could have partially offset the effects of high daytime temperatures.

In Europe, the colder temperatures prompted changes in the life-history of *P*. *clarkii*, causing a switch from multivoltine to uni- or bivoltine life cycles, and the onset of the breeding period to vary latitudinally with water temperature [58–59]. Indeed, recent studies suggest the limited niche conservatism in IAS may be a common mechanism driving their expansion [60–62], as acclimatization allows physiological function over wide temperature ranges [63–64]. Our results suggest that the low performance in the long heat wave may be correlated with the long-term acclimatization of *P*. *clarkii* to the relatively lower temperatures in Portugal, which is somewhat supported by the low survival of *P*. *clarkii* acclimated to 10°C when exposed to 30°C [65]. Furthermore, genetic adaptation over a short temporal scale has been documented in many species [66–68], and may have shifted the optimum temperature range or the overall shape of the temperature performance curve of *P*. *clarkii*. While *P*. *clarkii* may revert these adaptations to withstand longer, more frequent and intense heat waves, its distribution in Mediterranean climate regions may still contract [69], as heat waves will expose *P*. *clarkii* more often to drought in shallow water bodies. Nevertheless, the rise in winter minima projected for central and northern Europe may favor its expansion, as temperature is one of the main factors limiting *P*. *clarkii*’s distribution [70].

## Conclusions

Our results support those from other taxonomic groups, suggesting that temperature modulates the nutrient intake and the feeding preferences of omnivorous ectotherms, illustrating how these may develop under new climates. Given the positive effects on growth of the assimilation of plant material at higher temperatures, it is possible that diet regulation may help *P*. *clarkii* to cope with increased heat stress from longer heat waves in the future. While unlikely to boost *P*. *clarkii*’s Mediterranean populations, the changes projected in the severity, frequency and duration of heat waves may alter its trophic niche, especially in the juvenile stage, aggravating both its ecological impact on aquatic vegetation and economic damage to rice fields. This crayfish is known to act as a keystone species and changes in its functional role may echo through the whole food web, opening new trophic pathways or closing previously existing ones, possibly affecting its nutritional value to predators, its predatory impact upon macroinvertebrates and its grazing impact upon macrophytes [71]. Such modifications may even reshape the ecosystem function of the freshwaters inhabited by this alien invader, as macrophyte consumption by *P*. *clarkii* has been linked to shifts to alternative stable states with turbid water [72]. Finally, the tendencies in our results with *P*. *clarkii* suggest that the climatic changes projected for the near future may potentially drive significant changes in the most predominant impacts of other invasive alien species and warrants monitoring of the world’s ectothermic invaders and their consumptive impacts.

## Supporting information

S1 Table Data set of the juvenile *Procambarus clarkii*. Biometrics and isotope data.
(PDF)

S2 Table Data set of the adult *Procambarus clarkii*. Biometrics and isotope data.
(PDF)
