# Supplementary material for: Can heat waves change the trophic role of the world’s most invasive crayfish? Diet shifts in Procambarus clarkii
Source: PLoS One. 2017 Sep 5;12(9):e0183108. doi: 10.1371/journal.pone.0183108 (PMC5584761; doi:10.1371/journal.pone.0183108)
Supplement: S1 Table — Biometrics and isotope data. (PDF) [file pone.0183108.s001.pdf]

ID	Temperature	Diet	Sex	POCL (mm)	Growth (mg/day)	Body reserves (%)	Fulton's Index	d13C	d15N	%C	%N
901	C	Animal	F	17,66	4,51	6,71	0,22	-26,56	8,38	40,08	13,37
115	C	Animal	F	15,55	8,36	3,66	0,19	-25,56	8,24	38,00	12,83
140	C	Animal	F	15,94	6,30	6,08	0,23	-26,81	8,61	39,95	13,09
167	C	Animal	F	16,01	5,75	7,48	0,22	-26,16	8,52	39,44	12,98
35	C	Animal	F	16,96	4,11	6,67	0,22	-27,34	8,95	42,06	13,80
116	C	Animal	M	14,15	-5,07	5,13	0,19	-26,00	8,57	40,67	13,26
159	C	Animal	M	15,01	0,96	5,78	0,20	-26,44	8,31	40,24	13,20
45	C	Animal	M	15,90	4,93	6,37	0,23	-26,44	8,65	43,43	14,09
126	C	Animal	M	15,67	3,42	5,00	0,24	-27,11	8,84	41,88	13,67
158	C	Animal	M	16,88	5,34	6,23	0,22	-27,77	9,11	39,80	12,95
166	C	Mixed	F	17,92	1,92	6,29	0,19	-27,05	8,52	39,28	12,84
56	C	Mixed	F	15,34	1,23	6,32	0,20	-27,03	8,50	40,16	12,89
104	C	Mixed	F	17,15	-0,27	5,91	0,20	-25,83	8,31	40,16	13,24
142	C	Mixed	F	16,73	5,07	6,99	0,20	-25,51	8,43	40,64	13,17
19	C	Mixed	F	15,67	8,36	5,96	0,21	-26,90	8,56	40,15	13,36
110	C	Mixed	M	17,53	-0,14	5,67	0,20	-27,35	8,28	40,90	13,40
36	C	Mixed	M	16,08	7,67	7,11	0,24	-25,39	8,06	42,87	13,51
78	C	Mixed	M	15,66	9,86	6,41	0,22	-27,66	8,85	41,01	13,65
131	C	Mixed	M	16,71	5,48	5,49	0,23	-27,38	8,84	41,46	13,40
163	C	Mixed	M	14,43	4,25	5,46	0,24	-26,17	8,90	42,87	13,99
120	C	Plant	F	14,17	-0,96	3,15	0,20	-28,17	9,21	38,03	12,62
49	C	Plant	F	16,43	3,42	5,15	0,18	-26,54	8,92	41,69	13,25
76	C	Plant	F	15,54	3,15	6,05	0,20	-28,42	8,90	41,09	13,28
86	C	Plant	F	15,72	4,79	4,56	0,21	-26,18	8,91	46,82	15,23
106	C	Plant	F	17,32	1,37	3,86	0,21	-27,62	8,63	38,97	12,92
139	C	Plant	M	18,21	-2,74	4,69	0,20	-28,89	9,32	38,62	12,76
41	C	Plant	M	16,56	-0,41	4,41	0,19	-27,86	8,63	40,18	13,02
179	C	Plant	M	14,57	3,70	3,78	0,20	-26,86	8,87	40,83	13,35
18	C	Plant	M	15,68	-2,47	4,35	0,20	-28,42	9,44	40,88	13,48
132	C	Plant	M	16,77	3,84	5,20	0,22	-27,61	8,42	40,21	12,91
183	NS	Animal	F	14,10	7,67	4,95	0,23	-25,93	8,77	39,72	13,30
50	NS	Animal	F	14,85	6,71	6,64	0,22	-26,63	8,43	36,30	11,98
128	NS	Animal	F	16,93	7,95	4,39	0,27	-26,82	8,67	41,88	14,05
124	NS	Animal	M	14,70	9,04	4,05	0,17	-26,42	9,36	41,33	13,77
8	NS	Animal	M	17,70	8,63	4,03	0,23	-27,38	8,64	39,85	13,08
186	NS	Mixed	F	15,70	11,10	4,08	0,19	-26,78	8,61	41,23	13,68
21	NS	Mixed	F	15,19	4,11	4,41	0,21	-27,29	8,64	41,67	13,58
40	NS	Mixed	F	17,79	25,58	5,52	0,23	-27,06	8,11	42,50	13,70
75	NS	Mixed	F	15,69	9,18	5,10	0,21	-25,90	7,60	41,55	12,88
113	NS	Mixed	F	13,56	5,48	5,36	0,19	-26,12	7,75	39,68	13,18
97	NS	Mixed	M	17,04	14,52	3,35	0,27	-26,23	8,17	41,10	13,53
68	NS	Mixed	M	15,85	16,58	3,98	0,23	-26,62	8,29	41,48	13,44
65	NS	Mixed	M	16,47	14,52	4,91	0,23	-25,91	8,19	39,90	13,40
20	NS	Mixed	M	13,27	0,38	4,84	0,21	-25,44	7,81	36,25	11,79
908	NS	Mixed	M	15,94	14,90	4,71	0,21	-26,70	9,44	41,15	13,10
73	NS	Plant	F	15,94	2,47	5,31	0,22	-27,51	9,37	45,40	14,96
9	NS	Plant	F	16,15	2,47	4,17	0,21	-29,50	9,71	38,95	12,99
160	NS	Plant	F	16,55	15,75	4,44	0,21	-27,63	9,25	34,01	10,94

165	NS	Plant	F	15,26	3,97	4,04	0,20	-27,92	10,12	38,68	12,48
12	NS	Plant	F	17,24	5,34	5,39	0,22	-28,41	9,22	41,73	13,54
112	NS	Plant	M	15,38	8,90	3,53	0,21	-28,10	8,84	38,43	12,79
62	NS	Plant	M	17,29	-1,23	5,60	0,22	-28,95	9,45	39,18	12,68
150	NS	Plant	M	15,76	-1,92	4,97	0,18	-28,31	9,45	39,05	12,51
83	NS	Plant	M	15,82	-3,56	2,94	0,18	-28,40	9,69	39,76	12,94
82	NS	Plant	M	15,19	2,88	4,93	0,23	-28,01	9,21	40,65	12,73
144	SHW	Mixed	F	16,91	6,58	4,24	0,23	-26,09	8,40	39,63	12,80
29	SHW	Mixed	F	16,36	13,84	4,96	0,22	-25,82	7,73	40,26	12,96
69	SHW	Mixed	F	16,73	13,01	4,58	0,23	-27,49	9,06	40,20	13,46
180	SHW	Mixed	F	14,26	12,05	5,15	0,23	-25,37	8,35	42,08	14,05
61	SHW	Mixed	F	17,40	8,46	5,12	0,20	-26,96	8,44	41,21	13,73
138	SHW	Mixed	M	16,52	8,77	4,10	0,19	-26,52	8,44	41,38	12,83
111	SHW	Mixed	M	15,83	3,70	4,76	0,22	-26,24	8,73	42,50	13,61
42	SHW	Mixed	M	17,28	12,19	5,03	0,22	-26,88	8,97	39,49	13,01
64	SHW	Mixed	M	16,39	9,45	6,73	0,22	-26,91	8,97	39,39	13,40
85	SHW	Mixed	M	13,11	8,08	3,13	0,20	-25,75	8,36	41,10	13,21
95	SHW	Plant	F	16,45	-0,55	3,90	0,18	-29,36	9,86	39,22	12,46
168	SHW	Plant	F	15,45	2,60	2,76	0,21	-27,38	9,43	39,77	12,90
175	SHW	Plant	F	15,59	-1,64	5,76	0,18	-29,30	9,11	40,91	13,45
7	SHW	Plant	F	16,83	1,23	4,51	0,23	-28,78	9,88	40,23	12,95
34	SHW	Plant	F	17,23	4,38	4,79	0,22	-29,17	10,02	42,10	13,33
151	SHW	Plant	M	15,46	-1,78	3,47	0,20	-28,25	8,99	40,24	13,45
47	SHW	Plant	M	15,27	5,89	4,87	0,24	-27,04	8,71	38,80	12,10
162	SHW	Plant	M	15,79	2,47	4,43	0,22	-28,94	9,00	39,97	12,85
130	SHW	Plant	M	16,97	2,19	4,10	0,21	-28,67	9,53	37,10	11,84
37	SHW	Plant	M	16,48	0,00	3,77	0,21	-27,70	9,59	40,24	12,82
903	LHW	Animal	F	18,72	0,00	4,31	0,21	-28,93	9,30	31,10	10,14
915	LHW	Animal	F	18,03	3,53	4,20	0,21	-27,08	9,17	41,62	13,99
907	LHW	Animal	M	18,40	0,59	5,38	0,20	-28,05	9,28	40,17	13,31
911	LHW	Animal	M	19,53	3,53	5,09	0,23	-27,41	8,84	41,22	13,78
912	LHW	Animal	M	18,53	0,98	3,79	0,22	-26,36	8,95	40,60	13,79
25	LHW	Mixed	F	17,92	10,19	5,38	0,19	-27,04	8,78	39,63	13,22
149	LHW	Mixed	F	15,71	7,26	5,06	0,17	-25,98	7,96	38,92	12,68
90	LHW	Mixed	F	12,42	9,32	5,21	0,21	-26,08	8,32	33,80	11,33
43	LHW	Mixed	F	16,84	9,73	4,15	0,19	-26,25	8,82	41,80	14,09
72	LHW	Mixed	F	15,43	9,04	5,93	0,19	-27,19	8,97	41,49	13,71
117	LHW	Mixed	M	15,43	15,21	5,96	0,20	-26,66	9,10	40,13	12,91
4	LHW	Mixed	M	17,31	11,78	4,43	0,18	-26,11	8,70	38,88	12,70
79	LHW	Mixed	M	14,16	7,88	5,29	0,18	-26,31	8,06	41,47	13,04
914	LHW	Mixed	M	14,08	1,37	3,73	0,18	-26,30	8,53	40,59	13,35
152	LHW	Mixed	M	14,43	9,72	5,58	0,22	-26,02	8,16	37,06	11,92
33	LHW	Plant	F	16,52	1,78	4,82	0,20	-28,12	9,25	39,55	12,98
30	LHW	Plant	F	16,00	2,60	3,98	0,19	-28,36	9,48	38,97	12,89
46	LHW	Plant	F	17,31	-0,68	4,91	0,20	-27,05	9,36	40,00	12,96
57	LHW	Plant	F	17,44	-3,29	4,76	0,19	-28,47	9,62	38,90	12,70
51	LHW	Plant	F	14,29	2,47	5,52	0,18	-27,83	9,53	39,07	12,99
80	LHW	Plant	M	16,22	-1,64	5,10	0,17	-26,65	9,68	40,06	12,46
127	LHW	Plant	M	15,63	-0,55	3,65	0,19	-28,54	9,40	40,01	13,25
100	LHW	Plant	M	17,57	2,33	4,50	0,21	-27,67	8,98	40,85	12,72

10	LHW	Plant	M	16,91	-4,79	2,14	0,16	-29,30	9,62	37,74	12,21
16	LHW	Plant	M	15,27	2,74	3,90	0,21	-28,86	9,29	39,17	12,97
