# Supplementary material for: Can heat waves change the trophic role of the world’s most invasive crayfish? Diet shifts in Procambarus clarkii
Source: PLoS One. 2017 Sep 5;12(9):e0183108. doi: 10.1371/journal.pone.0183108 (PMC5584761; doi:10.1371/journal.pone.0183108)
Supplement: S2 Table — Biometrics and isotope data. (PDF) [file pone.0183108.s002.pdf]

ID	Temperature	Diet	Sex	POCL (mm)	Growth (mg/day)	Body reserves (%)	Fulton's Index	d13C	d15N	%C	%N
1001	C	Plant	M	24,60	-5,42	1,08	0,19	-26,94	9,22	40,52	12,83
1013	C	Plant	F	34,65	-14,92	3,71	0,28	-25,95	7,11	40,25	12,09
1025	C	Plant	M	32,44	-32,37	2,27	0,27	-26,99	12,13	40,24	11,93
1042	C	Plant	F	30,98	-15,25	4,38	0,25	-26,76	9,89	41,05	13,11
1050	C	Plant	F	28,97	-18,31	4,21	0,23	-26,04	9,55	39,60	12,75
1125	C	Plant	F	31,97	-29,66	3,66	0,24	-26,99	7,80	40,65	12,20
1136	C	Plant	M	28,70	-18,31	1,66	0,25	-27,56	9,75	38,49	12,03
1004	C	Animal	M	23,70	11,53	2,13	0,22	-26,40	11,30	37,93	12,41
1019	C	Animal	F	34,64	-1,36	2,18	0,30	-26,84	8,11	41,12	12,27
1068	C	Animal	F	32,74	-14,24	1,69	0,22	-24,88	9,37	38,49	12,36
1092	C	Animal	F	29,66	-21,86	9,75	0,24	-26,10	7,68	41,93	12,12
1098	C	Animal	M	29,09	-20,68	1,96	0,26	-25,13	8,69	42,06	12,41
1099	C	Animal	F	27,21	-19,15	2,10	0,24	-25,29	11,40	40,90	12,57
1033	C	Mixed	F	33,30	-23,39	7,24	0,24	-25,06	8,80	43,91	12,67
1072	C	Mixed	M	30,88	-25,25	2,00	0,31	-25,54	6,45	41,05	12,37
1074	C	Mixed	F	30,94	-19,15	5,58	0,23	-27,83	10,07	42,22	12,76
1077	C	Mixed	M	28,38	-15,42	3,48	0,24	-27,19	10,13	40,06	12,94
1084	C	Mixed	M	29,89	-11,69	2,79	0,21	-26,62	9,44	41,11	13,46
1085	C	Mixed	F	29,32	-28,64	3,97	0,23	-24,54	10,44	40,58	12,60
1103	C	Mixed	F	32,20	-23,90	3,16	0,24	-26,24	11,26	35,86	11,27
1012	NS	Plant	M	29,53	-27,29	2,08	0,25	-24,65	8,99	39,23	11,43
1037	NS	Plant	M	30,96	-19,15	2,49	0,25	-25,51	9,36	41,29	11,85
1071	NS	Plant	F	33,97	-48,14	4,27	0,20	-26,24	9,05	38,91	11,87
1081	NS	Plant	F	28,94	-16,44	4,59	0,24	-24,46	7,86	42,37	12,99
1105	NS	Plant	F	32,75	-10,34	4,16	0,29	-25,91	6,81	39,23	12,13
1117	NS	Plant	M	26,58	-10,68	3,05	0,23	-25,30	9,58	41,07	13,10
1119	NS	Plant	F	31,83	-16,27	4,23	0,24	-26,45	9,30	40,59	12,88
1003	NS	Animal	F	31,67	-8,14	11,33	0,22	-25,60	9,55	39,39	11,67
1010	NS	Animal	M	31,09	-35,93	1,70	0,26	-24,89	9,08	36,72	10,62
1043	NS	Animal	F	30,07	-24,24	3,67	0,24	-22,74	10,87	40,04	12,98
1066	NS	Animal	F	31,56	-21,19	3,30	0,25	-24,27	9,12	40,53	13,13
1090	NS	Animal	M	30,11	-10,18	1,33	0,24	-25,75	10,03	41,42	13,05
1014	NS	Animal	F	33,59	-26,55	2,52	0,25	-25,26	9,06	41,32	13,10
1134	NS	Animal	F	31,04	23,28	3,09	0,24	-27,39	8,22	40,79	13,20
1048	NS	Mixed	F	30,36	-29,83	3,61	0,22	-25,05	9,64	38,82	12,13
1052	NS	Mixed	F	33,92	-26,95	8,05	0,24	-25,75	7,61	43,49	13,33
1054	NS	Mixed	F	31,89	-27,80	3,00	0,24	-25,61	10,42	41,36	13,20
1080	NS	Mixed	M	28,26	-25,42	2,29	0,26	-25,11	9,51	41,48	13,26
1082	NS	Mixed	M	30,19	-16,95	2,62	0,27	-25,43	7,61	42,51	13,10
1115	NS	Mixed	F	28,82	-11,02	4,72	0,23	-27,58	9,86	40,85	12,47
1128	NS	Mixed	M	25,36	-5,93	3,85	0,26	-25,85	10,21	41,45	12,47
1034	SHW	Plant	F	32,09	-2,03	2,30	0,30	-26,31	8,18	37,18	11,01
1038	SHW	Plant	F	29,72	-42,71	4,98	0,23	-23,71	8,60	42,30	12,92
1049	SHW	Plant	M	27,79	-27,12	3,64	0,22	-25,75	9,88	41,51	13,56
1065	SHW	Plant	F	33,49	-16,44	3,65	0,25	-25,14	8,00	42,15	13,55
1083	SHW	Plant	M	29,39	-17,97	1,27	0,26	-24,75	9,38	38,74	12,02
1101	SHW	Plant	M	34,26	-39,66	2,61	0,25	-25,81	9,16	38,97	12,58

1130	SHW	Plant	F	26,09	-5,25	4,77	0,24	-25,25	7,39	42,46	13,09
1002	SHW	Animal	F	32,69	-42,20	9,24	0,24	-24,26	8,63	38,34	11,90
1017	SHW	Animal	F	34,10	-31,86	4,98	0,21	-25,05	9,52	41,27	13,37
1018	SHW	Animal	M	33,32	-18,31	2,91	0,24	-26,26	8,88	42,35	12,86
1036	SHW	Animal	F	30,43	-36,61	3,90	0,22	-24,33	9,66	39,48	13,36
1055	SHW	Animal	M	32,86	-45,93	2,95	0,26	-26,66	10,01	40,21	13,36
1113	SHW	Animal	M	29,97	-11,86	2,69	0,22	-27,30	9,51	41,61	14,09
1097	SHW	Animal	F	31,21	-23,97	2,75	0,24	-27,14	8,68	43,13	13,72
1008	SHW	Mixed	F	27,64	9,66	4,40	0,22	-25,78	10,42	42,39	13,85
1044	SHW	Mixed	F	30,69	-30,34	3,14	0,21	-25,61	8,43	40,58	13,17
1061	SHW	Mixed	F	32,96	-5,93	5,62	0,22	-24,61	9,16	40,87	13,30
1062	SHW	Mixed	F	32,73	-23,05	6,30	0,25	-25,15	8,10	42,24	13,11
1100	SHW	Mixed	M	28,80	-27,12	3,08	0,27	-25,96	9,59	41,20	13,55
1107	SHW	Mixed	M	31,29	-26,10	3,49	0,27	-25,71	8,19	41,06	13,26
1032	SHW	Mixed	M	31,49	-26,38	1,04	0,20	-23,15	9,19	40,36	12,32
1006	LHW	Plant	M	28,27	-38,64	4,54	0,17	-25,84	9,92	39,74	12,58
1021	LHW	Plant	M	33,21	-53,22	0,99	0,26	-26,14	9,71	39,07	12,59
1035	LHW	Plant	F	33,55	-52,71	3,84	0,19	-26,24	8,75	38,73	12,19
1059	LHW	Plant	M	34,14	-49,15	1,52	0,29	-28,59	11,99	38,27	12,45
1095	LHW	Plant	F	30,07	-18,64	4,26	0,32	-24,29	9,84	41,37	12,86
1110	LHW	Plant	F	31,80	-34,07	3,45	0,21	-26,89	8,33	38,93	12,17
1118	LHW	Plant	F	25,16	-12,37	3,09	0,25	-26,60	7,86	40,27	12,62
1053	LHW	Animal	F	34,96	-57,63	3,51	0,23	-25,10	9,11	40,44	12,66
1045	LHW	Animal	M	30,16	-18,98	2,10	0,26	-23,72	9,67	41,18	12,79
2161	LHW	Animal	F	27,08	-68,91	3,46	0,23	-25,59	9,23	37,95	2,44
1022	LHW	Mixed	F	34,26	-70,68	3,84	0,18	-25,62	8,40	39,69	12,66
1051	LHW	Mixed	F	31,27	-35,93	4,07	0,21	-26,57	9,24	38,18	12,26
1058	LHW	Mixed	M	32,15	-31,02	1,99	0,27	-25,76	7,94	42,23	13,11
1104	LHW	Mixed	F	32,55	-48,14	3,78	0,22	-27,10	10,10	39,18	12,33
1106	LHW	Mixed	F	34,80	-36,27	6,57	0,22	-24,78	9,11	42,63	12,01
2178	LHW	Mixed	M	27,61	-17,53	3,45	0,26	-24,91	8,88	38,33	11,76
2185	LHW	Mixed	M	28,95	-24,11	3,42	0,22	-25,98	8,83	42,59	13,32
